# Nitrous Oxide

**Published:** 1865-10

**Authors:** 


### Selections.

NITROUS OXIDE—IS IT SAFE FOR INHALING.—*Communicated for the Boston Medical and Surgical Journal.*—This is a question often asked by the patient about to inhale it for anæsthetic or sanitary purposes.

To this query all varieties of answers are returned by all grades of intelligence. One "professor," who claims a wide scope in the field of industrial chemistry, basing his opinions on the rude experiments of Davy and the older chemists, pronounces it a dangerous inhalant, of which the public should beware. Another "professor," equally assumptive, clamors for its indiscriminate use in hygiene and anæsthesia; and so opinions differ. The question is one we care not to discuss, farther than may be done in the simple statement of some of the experiments made by ourselves and one or two friends during the past year.

Every agent employed in medicine has its right and its wrong uses; and no drug wrongly prepared or ignorantly administered should be made responsible for its failure to do that claimed for it by the profession.

Few agents employed in surgery and hygiene are more widely known and less understood than nitrous oxide. The fact that it has been taken possession of to a great extent by charlatans in science, has caused the more intelligent to neglect the study of its nature and adaptation to the relief of pain and disease.

When properly prepared and properly administered, we can not but regard it among the safest of inhalants. That prepared by ourselves we have inhaled to entire anæsthesia *one hundred and ten* times during the past ten months, with a very perceptible improvement in our general health, while we have given this gas to others, for sanitary and anæsthetic objects, some three hundred times in the same period; and in no single instance do we remember meeting with the least unfavorable result. In certain dental establishments, in this city, it is almost, if not entirely, used for anæsthesia. The proprietor of one of these recently stated to us that it had been employed by him for anæsthesia in nearly eight thousand cases, with not a serious result.

But by some it is urged that while perhaps safe for brief anæsthesia, this can not be predicted of the gas in protracted operations. In at least three instances, we have known entire anæsthesia maintained during surgical operations lasting from four to seven minutes, with the most happy effects on awakening. Others assure us of success with this in cases of anæsthesia much more protracted. From what we have heard and witnessed ourselves, we have reason to believe that entire insensibility to pain may be safely maintained for a longer period than with ether or chloroform. This may be inferred from the vitalizing character of nitrous oxide, which maintains the temperature and pulse by oxidizing the blood, while it is well known that the effects of ether, or more particularly chloroform, are the reverse of this.

In the preparation of nitrous oxide and its indiscriminate use by ignorant and unskilled practitioners, is it any cause for wonder that cases of injury should be occasionally reported? Considering its extensive use, is it not rather a wonder that so few such should occur?

Until within a short period, insensibility was produced, in most of the dentists' offices where the gas was used, by inhaling this from small rubber bags and allowing the breath to return and be re-inhaled—the quantity of the nitrous oxide being in many cases wholly insufficient to counteract the poisonous carbonic acid from the lungs. Thus, insensibility was produced by the aid of asphyxia—the lips of the patient often assuming a purple hue, while the head seemed distended and filled with fearful sounds, followed by severe headache upon the patient's returning to consciousness. Such a barbarous application of this agent has only served to excite ridicule from certain caustic pens, and prejudice the community against the gas rather than against such a disgusting application. Indeed, even professors in some of our colleges and compilers of our text books on chemistry, have done not a little to mislead in this matter. In four school chemistries now before us, this gas is represented as being prepared by washing in a single bottle holding less than one quart of water, and in two of these books the inhalation is directed to be made from a *bladder of about the capacity of the patient's head!* With such guides, no wonder that itinerant professors have such success, and that in some cases dentists find it "injurious" to their patients.

To be safe, nitrous oxide, like chloroform, should be properly made and administered. In making this gas, we employ an automatic regulator of the heat, confining the nitrate of ammonia in the flask to a temperature scarcely above 400°. The results of decomposition at this point are quite different from those where the heat of the salt is fluctuating, rising at times to 500°, and even to 600°, as is common by the ordinary process of heating. Thus prepared, the gas in its passage to the gas-holder is forced through five one-and-a-half-gallon glass washers, filled with an alkaline solution and pure water; each washer being provided with perforated glass tubes for finely dividing and washing the gas. Thus five distinct plunges are made through as many feet of water, when the gas is passed into a zinc receiver, where it is confined over water. This whole process is simple, safe and economical.

For inhaling, we employ an improved valved apparatus, drawing the gas directly from the zinc holder and exhaling into the open air. With this arrangement, perfect anæsthesia in its most agreeable form may be produced, while even the most sensitive lungs suffer not the least inconvenience.

The efficiency of nitrous oxide depends on the degree of its absorption by the blood which meets it in the lungs; and this of course, on the amount inhaled and the time it is retained in these. We have seen anæsthesia caused by only three deep and slow inhalations of capacious lungs; while in the case of surface breathing from compressed or diseased lungs, the process is often prolonged to twenty or more inhalations. And in this connection we may say that in several instances we have found the capacity of the lungs increase with each trial of the gas, with a corresponding shortening of the period preceding anæsthesia.

As a sanitary agent in pulmonary consumption and certain acute diseases, we feel confident that nitrous oxide in its purity has its merits, sufficient, at least, to claim the investigation of professional skill. May it not have at least a trial by such, and a fair and scientific verdict be rendered? Its extensive use in hygiene and surgery demands this, rather than that it be left in unskilled hands, a butt for professional lampoons.

*Boston, Angust 17th,* 1865.

